# Halophilic and Non‐Halophilic Microbial Communities in Relation to Physico‐Chemical Characteristics of Salt Mine Air

**DOI:** 10.1111/1758-2229.70095

**Published:** 2025-04-30

**Authors:** Aleksandra Puławska, Jolanta Kalinowska, Michalina Rachubik, Dominika Drzewiecka, Luciana Albuquerque, Conceiçao Egas, Krzysztof Krawczyk, Maciej Manecki, Camille Locht, Magdalena Kowalewicz‐Kulbat

**Affiliations:** ^1^ Department of Mineralogy, Petrography and Geochemistry Faculty of Geology, Geophysics and Environmental Protection, AGH University of Kraków Kraków Poland; ^2^ Department of Immunology and Infectious Biology Institute of Microbiology, Biotechnology and Immunology, Faculty of Biology and Environmental Protection, University of Lodz Lodz Poland; ^3^ Department of Biology of Bacteria Institute of Microbiology, Biotechnology and Immunology, Faculty of Biology and Environmental Protection, University of Lodz Lodz Poland; ^4^ CNC‐UC—Center for Neuroscience and Cell Biology University of Coimbra, UC‐Biotech Cantanhede Portugal; ^5^ CIBB—Center for Innovative Biomedicine and Biotechnology University of Coimbra, UC‐Biotech Cantanhede Portugal; ^6^ Genoinseq—Next Generation Sequencing Unit, Biocant Cantanhede Portugal; ^7^ University of Lille, CNRS, Inserm, CHU Lille, Institut Pasteur de Lille U1019‐UMR9017‐CIIL‐Center for Infection and Immunity of Lille Lille France

## Abstract

Salt mines are often used for halotherapy against lung and skin diseases. In addition to salt, they also contain various types of microorganisms, which remain poorly characterised. Here, we examined culturable halophilic and non‐halophilic microbial populations in relation to the physico‐chemical characteristics in the air of four different sites of the Bochnia Salt Mine, a popular halotherapy resort in Southern Poland. At the mine entrance, the temperature was highest (20.8°C) and decreased with increasing distance from the entrance (15.5°C at 2671 m from entrance), while humidity increased from 55.9% to 77.0%, as did the NaCl concentration. At the entrance, non‐halophilic microorganisms prevailed, especially fungi that grew at 21°C. Halophiles gradually dominated with distance from the entrance, including halophilic archaea that grew at 28°C or 37°C on medium containing 15%, 20%, or 25% NaCl. Seven halophilic archaeal species were identified by 16S rRNA gene sequencing. The frequency of non‐halophiles was inversely related to distance from the entrance, humidity, and presence of ions, while the reverse was seen for halophiles. An exception was the site used for halotherapy, where non‐halophilic bacteria dominated. Thus, natural salt mines contain a wide variety of non‐halophilic and halophilic microorganisms, including archaea, which may contribute to the halotherapeutic effects.

## Graphical abstract

This study unveils the presence and densities of cultivable halophilic and non‐halophilic microorganisms present in the air sampled at four different sites of the Bochnia Salt Mine in Poland used for halotherapy. It highlights for the first time the presence of cultivable halophilic archaea in salt mine air in relation to the physico‐chemical characteristics of the aerosols and human activity during the summer season.

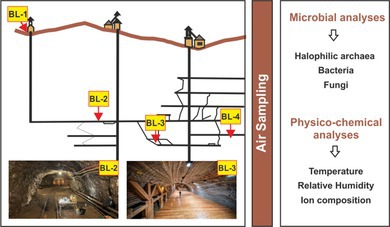

## 1 Introduction

Halotherapy is a popular adjunct treatment option and post‐treatment convalescence method for patients with respiratory diseases, especially diseases due to immune dysregulation, such as allergic asthma, chronic obstructive pulmonary disease, bronchitis, cystic fibrosis, as well as skin conditions and others (Chervinskaya and Zilber 1995). Several placebo‐controlled clinical studies have confirmed that salt mines improve the clinical state of patients with respiratory diseases (Gelardi et al. 2013; Bar‐Yoseph et al. 2017), providing further evidence to recommend halotherapy as an adjunct treatment (Hedman et al. 2006). However, other studies have not observed clinical improvement after use of a salt inhaler (Rabbani et al. 2013), suggesting that the therapeutic effects may vary according to the disease and/or the halotherapy environment (Lazarescu et al. 2014).

While exposure to NaCl‐saturated air has been shown to play a key role in the therapeutic effects (Sandu et al. 2015), the fact that studies carried out at different sites do not always report consistent findings (Rabbani et al. 2013) suggests that compounds other than NaCl may also contribute. In addition to NaCl, airborne particular matter in salt mines also contains other elements, the proportions of which may vary between different sites of the same salt mine, depending on the distance from the entrance and frequency of human presence (Pulawska et al. 2021).

In addition, salt mine aerosols may contain microorganisms, including bacteria and fungi (Gebarowska et al. 2018), and their composition can vary between different sites of the salt mines depending on the distance from the entrance and on human activities (Fraczek et al. 2013; Myszkowska et al. 2019). The presence of halophilic organisms, such as halophilic archaea, particularly well adapted for survival in high salinity, has not been reported yet. Several attempts to isolate halophilic archaea from the air of salt mines have failed (Vreeland et al. 1998; McGenity et al. 2000), although they are present in high amounts in various other hypersaline environments (Walsh et al. 2005; Schubert et al. 2010), including brines of salt mines. Halophilic archaea present in the air of salt mines may be of particular interest, as they may display immunomodulatory properties, as shown for species *Halorhabdus rudnickae* and *Natrinema salaciae* (Krawczyk et al. 2022), respectively isolated from a Wieliczka salt mine brine (Albuquerque et al. 2016) and the anoxic lake Medee (Albuquerque et al. 2012), and may therefore participate in the halotherapeutic effects.

Here, we investigated the physico‐chemical parameters and microbial composition, including halophilic archaea, of the air in four different sites of the Bochnia Salt Mine, one of the two Polish salt mines used for halotherapy. We compared the densities of halophilic microorganisms with those of non‐halophilic microorganisms in relation to the distance from the entrance, humidity, temperature, ion concentrations, and human activity. We concentrated on cultivable microorganisms to be able to study their biology and potential use as immunomodulatory agents or sources for novel anti‐microbial or anti‐cancer activities (Kowalewicz‐Kulbat et al. 2023) and other biotechnological applications (Oren 2010).

## 2 Materials and Methods

### 2.1 Sampling

**FIGURE 1:**
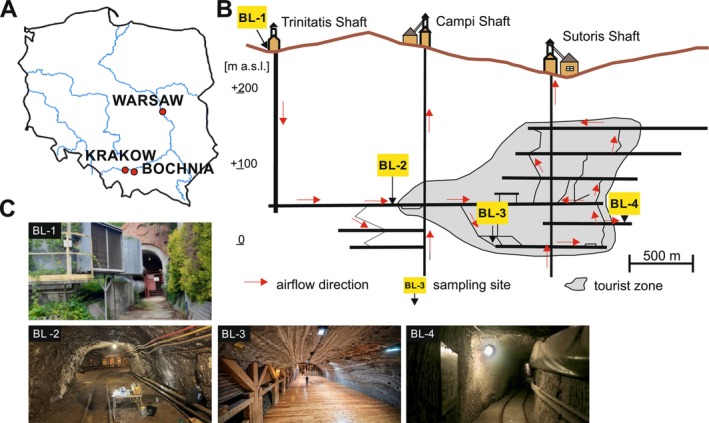
Location of the Bochnia Salt Mine and sampling sites. (A) Location of the Bochnia Salt Mine 40 km East of Krakow in southern Poland. (B) The four sampling sites of the Bochnia Salt Mine labelled BL‐1, BL‐2, BL‐3 and BL‐4. The red arrows indicate the air flow direction. The grey zone indicates the tourist and halotherapy zone. (C) Photographs of each of the sampling sites BL‐1, BL‐2, BL‐3 and BL‐4, as indicated.

The research was conducted in the Bochnia Salt Mine, located 40 km East of Kraków in southern Poland (Figure 1A). The mine, recognised as a UNESCO World Heritage site, ceased rock salt extraction in 1990. Since then, it has been repurposed for tourism, recreational activities, and therapeutic services (Puławska et al. 2021). The current spatial structure of the historic mine consists of 9 post‐mining galleries extending down to 350 m below the surface (Figure 1B). In July 2022, we sampled the air at four different locations in the order of increasing distance from the air intake of the mine (Figure 1B,C). The sampling strategy included a point at the air inlet (Trinitatis shaft) to provide a sample representative of the air pumped into the mine (BL‐1). This was followed by a series of sampling points located in underground passages at various distances from the air intake, both in areas closed to the public and in a mine chamber open to the public. BL‐2 is in an underground tunnel, 212 m below the ground surface and 1185 m from the entrance, which supplies air to the rest of the mine and is inaccessible to visitors. BL‐3 is located in the Ważyn Chamber used for halotherapy (248 m deep and 1728 m from the entrance), and BL‐4 is at a depth of 230 and 2671 m from the entrance, in an area not accessible to visitors.

At each location, air temperature and humidity were measured, as well as the chemical composition and the amounts of microorganisms. Two different methods were used to collect samples for chemical composition analysis of aerosols: the dry (filter) method and the wet (scrubber) method. Samples were collected in triplicate at each sampling site. For methodological details of the sampling procedures, please refer to the Supporting Information.

### 2.2 Chemical Analysis of Aerosols

The concentration of cations (Na^+^, NH_4_^+^, Mg^2+^, K^+^, Ca^2+^, Fe^3+^, Mn^2+^, Al^3+^, Ti^4+^, As^3+^, Ba^2+^, Cr^3+^, Cu^2+^, Mo^6+^, Ni^2+^, Pb^2+^, Zn^2+^) in air samples collected by the wet method was analysed by inductively coupled plasma mass spectrometry (ICP‐MS; ELAN 6100; PerkinElmer), while the concentration of anions (F^−^, Cl^−^, Br^−^, PO_4_^3−^, SO_4_^2−^) was analysed by ion chromatography (IC; ICS‐1100 Thermo Scientific). The concentration of TSP (solid particles) was measured gravimetrically by weighing filters. Water‐soluble cations (Na^+^, NH_4_^+^, Mg^2+^, K^+^, Ca^2+^) and anions (F^−^, Cl^−^, Br^−^, PO_4_^3−^, SO_4_^2−^, NO_2_^−^, NO_3_^−^) were extracted from filters and analysed by IC, while organic carbon (OC) and elemental carbon (EC) were analysed using the thermal‐optical method. Microelements (Al, Fe, Mn, Ti, As, Ba, Cr, Cu, Mo, Ni, Pb, Sr., Zn) were extracted via acid digestion and analysed using ICP‐MS. A detailed description of analytical procedures and detection limits for applied chemical analyses is presented in (Puławska et al. 2021).

### 2.3 Isolation and Culture Conditions

TSA and HBM‐agar plates with 15%, 20%, or 25% NaCl were incubated at 21°C, 28°C or 37°C under humid conditions for up to 10 days for non‐halophiles and for up to 3 months for halophiles. After incubation, the numbers of CFU were calculated and expressed as CFU/m^3^ of air. Based on the morphological characteristics of the colonies growing on HBM agar, single colonies with reddish pigments were cultured on fresh HBM agar with the same NaCl concentration as the initial plates until pure colonies were obtained. The pure isolates were frozen in 50% glycerol and stored at −80°C until used for molecular studies.

### 2.4 Genomic DNA Extraction

To identify the isolated strains grown on HBM agar at the species level, genomic DNA was extracted using the Nielsen method (Nielsen et al. 1995). Briefly, colonies grown on HBM agar containing 15%–25% NaCl were collected, washed, and suspended in TES buffer. A lysozyme solution was added, followed by incubation at 37°C for 2 h. A guanidium thiocyanate and sodium *n*‐lauryl sarcosine solution was then added, and the samples were incubated for 10 min on ice, followed by RNase treatment for 1 h at 37°C. Proteinase K was then added, and after incubation for 1.5 h at 37°C, ammonium acetate was added, and the samples were incubated for 10 min on ice. Chloroform:isoamyl alcohol (24:1, v/v) was then added, and the samples were vigorously shaken and then centrifuged. The upper phase was collected, and the DNA was precipitated by adding isopropanol. After centrifugation, the pellet was washed with 70% ethanol and then dried at room temperature before resuspension in ultrapure water. The DNA was then stored at −20°C.

### 2.5 PCR Amplification and Sequencing of 16S rRNA Genes

The 16S rRNA gene was amplified by PCR as described (Albuquerque et al. 2024). For bacterial isolates, the primers used were 27F (5’‐GAGTTTGATCCTGGCTCAG‐3′) and 1525R (5’‐AGAAAGGAGGTGATCCAGCC‐3′). For archaea, the primers used were 21F (5’‐TTCCGGTTGATCCTGCCGGA‐3′ and 1492R (5’‐TACGGYTACCTTGTTACG‐3′). The 16S rRNA PCR products were purified with NZYGelpure (NZYtech) according to the manufacturer's protocol. The samples were stored at −20°C until used. The 16S rRNA gene sequence (~700 bp) was determined by Sanger sequencing (Stab Vida, Portugal). Based on the partial 16S rRNA gene sequences, the taxonomic affiliation of each isolate was determined using the online EzBioCloud database (Yoon et al. 2017).

### 2.6 Statistical Analyses

Statistical analyses were performed with the GraphPad Prism 7 and STATISTICA 12.0 PL programs. Data are expressed as means ± SD. Differences between samples were analysed by the variance Kruskal‐Wallis non‐parametric test and the Mann–Whitney U test. The correlation analyses were performed using Pearson's r or Spearman's correlation R coefficient depending on the normality of the distribution of the data. *p* values ≤ 0.05 were considered significant.

## 3 Results

### 3.1 The Physicochemical Properties of Air in the Salt Mine

**FIGURE 2:**
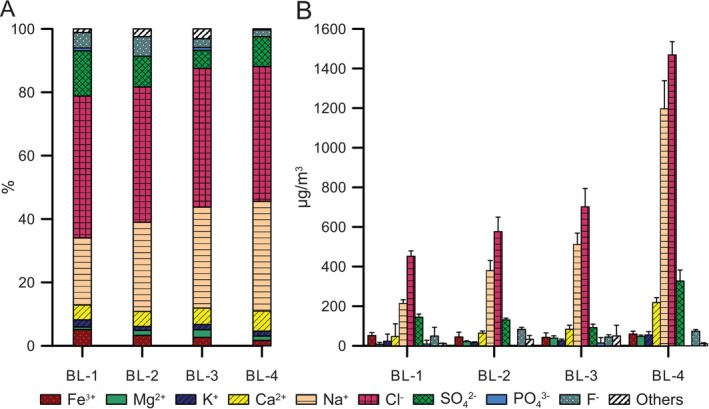
Ion composition in the liquid aerosol at the four sampling sites. The indicated ions were measured in the air at BL‐1, BL‐2, BL‐3, and BL‐4, as indicated, and expressed as relative proportions (%; A) and absolute numbers (μg/m^3^; B). Elements below the detection limit for all sampling events are not reported.

With increasing distance from the air inlet (from sampling points BL‐1 to BL‐4), the temperature gradually decreased from 20.8°C to 15.5°C, and the relative air humidity gradually increased from 55.9% to 77.0% (Table 1). The ion concentration in liquid aerosol also increased from BL‐1 (983 ± 190 μg/m^3^) to BL‐4 (3432 ± 285 μg/m^3^). This was largely due to increases in sodium and chloride (Figure 2A, Table S1). However, other ions, such as potassium, calcium, and sulfate, were also more abundant in BL‐4 compared to BL‐1. The amount of magnesium, although found in low quantities, also gradually increased from BL‐1 to BL‐4 (Figure 2B). Phosphate, aluminium, manganese, chromium, copper, and zinc were poorly abundant at any of the sampled sites and did not vary substantially according to the distance from the mine entrance. Ion concentrations in liquid aerosol solutions were several hundred times higher than water‐soluble ion concentrations in solid particles (Table S2).

**TABLE 1:** Environmental parameters of sampling sites.

	BL‐1	BL‐2	BL‐3	BL‐4
Distance from air entrance (m)	—	1185	1728	2671
Air relative humidity (%)	55.9	64.5	66.5	77.0
Air temperature (°C)	20.8	17.2	17.8	15.5

### 3.2 Microbial Composition of Air Samples

**FIGURE 3:**
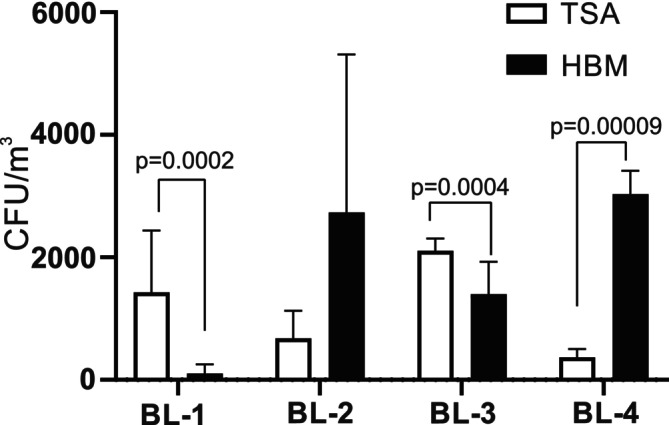
Presence of non‐halophilic and halophilic microorganisms in the air at the four sampling sites. Colonies of non‐halophilic microorganisms grown on TSA agar (white bars) and halophilic microorganisms grown on HBM agar containing 15%, 20% or 25% NaCl (black bars) were counted after growth for 10 days and 3 months, respectively, at 21°C, 28°C or 37°C and the four sampling sites. The values shown represent the average CFU counts +/− standard deviations at all relevant conditions, with three samples counted for each condition.

To study the relative proportions of halophilic microorganisms compared to other microorganisms in the four sampling sites, all microorganisms from 100 L of air were grown on TSA agar or HBM agar containing 15%, 20%, or 25% NaCl. Taking into account all temperatures and salt concentrations, BL‐1 was dominated by CFU counts on TSA agar, while BL‐2 and BL‐4 were dominated by CFU counts on HBM agar (Figure 3). A mixed profile slightly, but significantly dominated by CFU counts on TSA agar was found for BL‐3.

**FIGURE 4:**
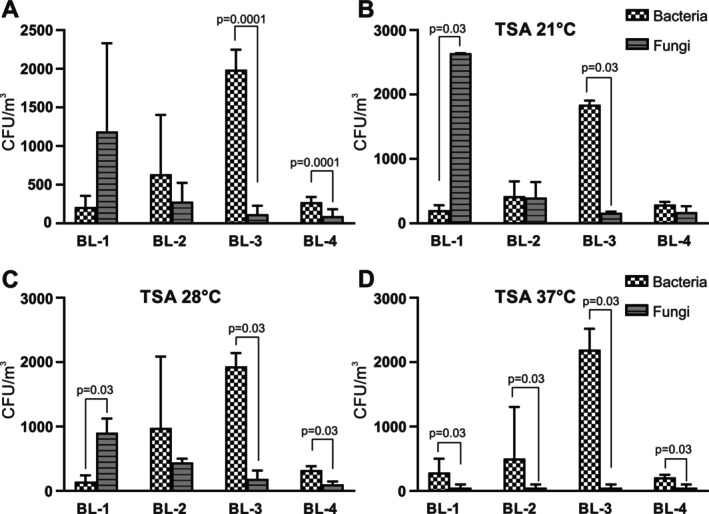
Presence of non‐halophilic fungi and bacteria in the air at the four sampling sites. Colonies of non‐halophilic bacteria (dotted bars) and fungi (hatched bars) collected from the air at the four indicated sampling sites were counted after growth for 10 days on TSA agar at 21°C, 28°C or 37°C. The values shown represent the average CFU counts ± standard deviations at all temperatures (A), or at 21°C (B), 28°C (C) or 37°C (D), with three samples counted for each condition.

When the samples collected on TSA agar were examined for the presence of mould fungi and bacteria, the fungi were found to dominate in BL‐1, while bacteria dominated in BL‐3 (Figure 4A). Mixed profiles were found in BL‐2 and BL‐4, although bacteria dominated slightly in both sites, which reached statistical significance only for BL‐4. When these samples were further subdivided according to their growth temperature, fungi in BL‐1 clearly dominated at 21°C (Figure 4B), while they became less prevalent at 28°C (Figure 4C) and at 37°C, where bacteria predominated (Figure 4D). In contrast, bacteria were more prevalent in BL‐3, regardless of growth temperature (Figure 4B–D). They were also more prevalent than fungi at 28°C and 37°C in BL‐2 and BL‐4.

### 3.3 Halophilic Microorganism Composition at the Four Sampling Sites

**FIGURE 5:**
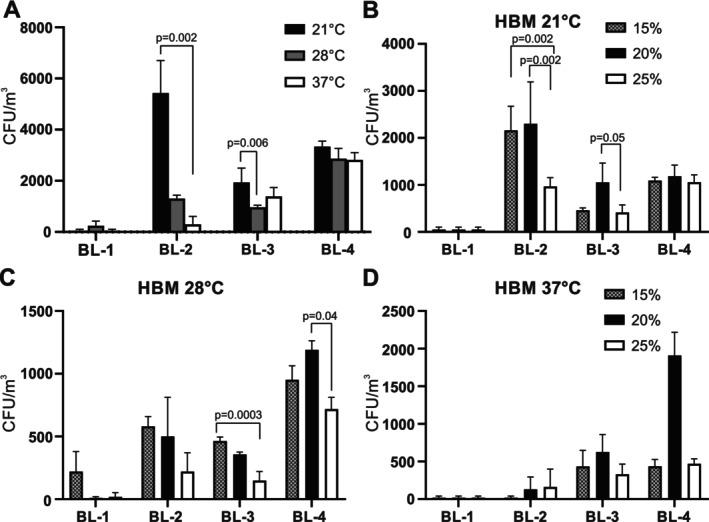
Presence of halophilic microorganisms in the air at the four sampling sites. Colonies of halophilic microorganisms collected from the air at the indicated sampling sites and grown on HBM agar containing 15%, 20% or 25% were counted after 3 months incubation at 21°C, 28°C or 37°C. The values shown in panel A represent the average CFU counts +/− standard deviations grown at all salinities at 21°C (black bars), 28°C (grey bars) or 37°C (white bars), with three samples counted for each condition. Values in panels B‐D represent the average CFU counts +/− standard deviations grown with 15% (grey bars), 20% (black bars) or 25°C (white bars) at 21°C (B), 28°C (C) or 37°C (D).

As expected, very few halophilic microorganisms collected in BL‐1 grew on HBM plates at any temperature tested (Figure 5A). Most halophiles from BL‐2 were recovered on HBM plates incubated at 21°C, followed by 28°C and then 37°C (Figure 5B–D). A more equal distribution was found for BL‐3 and BL‐4, although for BL‐3 the highest numbers of halophiles were also counted for plates incubated at 21°C. When the numbers of CFU of the halophiles were calculated according to salinity, most halophiles grew at 15% or 20% NaCl, especially in BL‐2 and BL‐3 at 21°C and 28°C (Figure 5B,C). For BL‐4 a more balanced profile was found, with the exception of plates incubated at 37°C, for which the numbers of CFU were found higher at 20% NaCl than at 15% or 25% (Figure 5D).

### 3.4 Correlation Between Physico‐Chemical Characteristics and Number of Halophile Isolates

**FIGURE 6:**
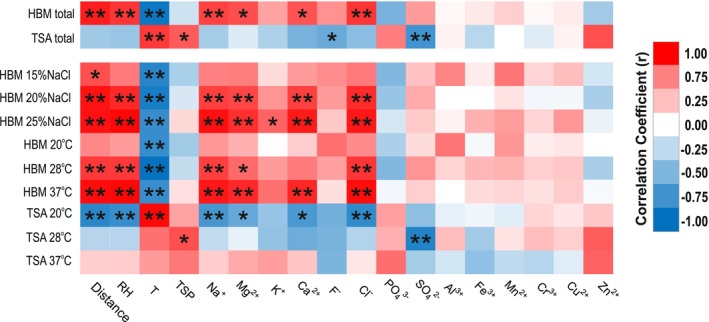
Correlation heat map of physico‐chemical characteristics and the microbiological profile at the four sampling sites. Correlations between physico‐chemical composition and halophilic/non‐halophilic microorganisms growing in different conditions are indicated by the colour code ranging from dark blue to dark red. Statistical significances are indicated by the stars. *, *p* < 0.05; **, *p* < 0.01. HBM, *Halobacterium* medium; TSA, Tryptic‐Soy Agar; RH, Relative humidity; T, temperature; TSP, Total suspended particulate matter.

In order to establish a potential link between the physico‐chemical characteristics at the four sampling sites with the presence of halophilic and non‐halophilic microorganisms, we compared temperature, humidity, and ion composition with the numbers of colonies isolated in each growth condition. A very strong positive correlation was found between the distance from the mine entrance, the relative humidity, and the numbers of colonies on HBM agar, especially on plates with 20% and 25% salinity grown at 28°C or 37°C, which corresponded to a very strong negative correlation with temperature (Figure 6). In contrast, growth on TSA medium, especially at 21°C, correlated strongly with increasing temperature but correlated negatively with increasing humidity. As expected, high salinity, especially high levels of sodium, magnesium, calcium, and chloride, correlated strongly with the numbers of microorganisms grown on HBM agar, especially those grown at 20% or 25% NaCl and at 28°C or 37°C. This was in contrast to microorganisms grown on TSA plates, especially at 21°C. A very strong negative correlation was also found between the presence of sulfate and the numbers of colonies on TSA plates, especially those grown at 28°C. The correlations with other ions, although globally positive for halophilic microorganisms and negative for non‐halophilic microorganisms, with the exception of zinc and phosphate, were less strong.

### 3.5 Identification of Halophilic Archaeal Species

As in this study we focused on halophilic archaea, we examined colonies, based on differences in colony morphology and growth on different salinities. Through 16S rRNA gene sequencing, we identified seven different archaeal species (Table 2). One species was isolated from site BL‐1, two from BL‐2, three from BL‐3, and two from BL‐4. Interestingly, the sequenced collection also contained one bacterial isolate ( *Lentibacillus persicus* ) recovered from site BL‐4, which formed a very light reddish colony.

**TABLE 2:** Halophilic microbial species isolated from the air of the Bochnia Salt Mine.

Sampling site	Halophilic species	Accession number	% identity	Domain	Media (% NaCl)
BL‐1	*Halococcus salifodinae* DSM 8989^T^	AOME01000075	(100%)^a^	A^b^	25%^c^
BL‐2	*Natrinema versiforme* XF10^T^	AB023426	(98.51%)	A	20%
BL‐2	*Halococcus morrhuae* DSM 1037^T^	AOMC01000054	(99.75%)	A	25%
BL‐3	*Halalkalicoccus paucihalophilus* DSM 24557^T^	LTAZ01000003	(99.10%)	A	15%
BL‐3	*Halococcus dombrowskii* H4^T^	AJ420376	(99.14%)	A	15%
BL‐3	*Halalkalicoccus subterraneus* GSM28^T^	MG097856	(99.64%)	A	15%
BL‐4	*Halococcus hamelinensis* 100A6^T^	AOMB01000011	(99.63%)	A	20%
BL‐4	*Lentibacillus persicus* AMB31^T^	FN376846	(99.90%)	B	20%

^a^ Percent of 16S rRNA gene identity with the closest species with validly published names using the online EzBioCloud database (Yoon et al. 2017).

^b^ A = archaea; B = bacteria.

^c^ Percent of salinity at which the strains were cultured on HBM agar.

## 4 Discussion

To the best of our knowledge, this is the first report on the isolation of viable, cultivable halophilic archaea from the air. Several previous attempts to collect such organisms from the air have failed (Vreeland et al. 1998; McGenity et al. 2000). By using an air sampling device directly connected to agar plates containing the appropriate medium, we sampled here the air at four different sites of the Bochnia Salt Mine and compared the density of halophilic and non‐halophilic microorganisms according to the distance from the mine entrance. We focused this study on cultivable microorganisms with special attention to halophilic archaea, since they are an important potential source of novel compounds for therapeutic and biotechnological applications (Kowalewicz‐Kulbat et al. 2023; Oren 2010). We also compared microbial densities to the physico‐chemical characteristics at each site.

At the mine entrance, mostly non‐halophilic organisms were detected, with a major contribution of mould fungi, and only relatively few halophiles were isolated at this site. Sodium, chloride, and sulfate were the dominant ions in the air at this site. The dominance of fungi was expected, as the samples were collected in the summer season, when fungal spores are present in high densities in the outdoor air. It will be interesting to see whether this profile changes seasonally.

Deeper into the salt mine, in an area that is not accessible to the public, the ion concentrations increased in the air, but remained dominated by sodium, chloride, and sulfate. Here, halophiles dominated over other microorganisms. They reached densities of several thousand per square meter.

The density of non‐halophilic microorganisms increased again at the third location, the Ważyn chamber, used for recreational purposes and halotherapy. This is the largest chamber in the mine, measuring over 250 m in length (Pulawska et al. 2021). It contains sports courts for futsal, basketball, and volleyball, a playground, a relaxation area, and a designated sleeping zone with accommodations for up to 250 people. It is the primary site for halotherapy sessions, which include both overnight stays (lasting approximately 13 h) and daily 3‐h inhalation sessions. Furthermore, all guided tours in the mine conclude with a minimum 30‐min stay in the Ważyn Chamber, providing visitors with additional exposure to its unique microclimatic conditions. Halophiles were also present on this site, albeit at a slightly lower density than at the previous site. Halophiles dominated again at the fourth sampling site, at 2671 m from the entrance, not accessible to visitors. This site had also the highest ion concentration, composed mainly of sodium, chloride, sulfate, and calcium. It had also the highest degree of humidity and the lowest air temperature.

The high density of non‐halophilic fungi at the mine entry and of bacteria in the Ważyn chamber strongly suggest that these organisms originated from the outside environment and human activity, respectively, while the halophiles most likely came from aerosolized rock material or salt present in the mine at locations distant from the entrance. Although this is the first report on culturable archaea in air samples, the presence of archaea in outdoor and indoor aerosols has been suggested in several studies based on DNA sampling, PCR amplification, and 16S rRNA gene sequencing (Rastmanesh et al. 2024). Air samples collected in Beijing during springtime yielded from one to 1000 16S rRNA gene copies per square meter, depending on the degree of air pollution (Niu et al. 2021). Some studies, such as those performed in swine confinement buildings, showed high concentrations of archaeal DNA, of up to 10^8^ 16S rRNA gene copies per cubic meter of air (Nehmé et al. 2009). While culture‐independent methods such as 16S rRNA gene copy measurements are useful for metagenomic purposes, they provide no information on the densities of viable archaea in the air, which are probably much lower in numbers than 16 rRNA gene quantitation would suggest. In this study, we found culturable halophilic archaea densities of several thousand per cubic meter in three different locations of the Bochnia Salt Mine, at a distance of at least 1 km from the mine entrance. Live fungi and bacteria had already been isolated from Polish salt mines (Gebarowska et al. 2018; Fraczek et al. 2013). This report shows that numerically halophilic archaea significantly exceed bacteria and especially fungi when sampled beyond the entrance of the mine. This was particularly evident in locations not open to the public.

Seven different halophilic archaeal species and one halophilic bacterial species were isolated. In contrast, in swine confinement buildings, the archaeal diversity was found to be very low (Nehmé et al. 2009). Most of these archaea were anaerobic and methanogenic, most likely originating from aerosolized swine manure. Methanogenic archaeal 16S rRNA genes have also been detected in aerosols from poultry operations, and differences between cage‐housed and floor‐housed operations have been documented, with significantly higher amounts in cage‐housed poultry operations (Just et al. 2013). Intriguingly, workers from cage‐housed poultry farms were more often affected by respiratory symptoms than workers from floor‐housed farms, suggesting an inverse relationship between the presence of methanogenic archaea and respiratory health. In a mouse model of hypersensitivity pneumonitis, some methanogenic archaeal species, present in high concentrations in aerosols of dairy farms, were indeed found to induce typical immunopathological features in the lungs after intranasal administration (Lecours et al. 2011).

In this study, we did not collect methanogenic archaea, as they are mostly strict anaerobes, and we cultured the archaea in the presence of oxygen. However, in contrast to bioaerosols in agricultural facilities thought to be associated with respiratory diseases, exposure to aerosols in salt mines is associated with therapeutic effects against lung diseases. It is therefore possible that halophilic archaea found in salt mines, in contrast to methanogenic archaea found in dairy farm aerosols, may be beneficial for the treatment and prevention of lung diseases. We have previously shown that at least two species of halophilic archaea are able to direct human dendritic cells towards a Th1‐type profile (Krawczyk et al. 2022), known to be associated with the prevention of allergic asthma. Studies are currently underway to examine the effect of the different halophilic archaeal species isolated from the air of the Bochnia Salt Mine on immune cells. Strong immunomodulatory effects of some of these halophiles on immune cells, in particular on antigen‐presenting cells, such as dendritic cells, and other innate immune cells may potentially participate in the halotherapeutic effects. It may also be interesting to focus future studies on the effect of these strains on immune cells from patients with specific conditions, such as patients with allergic asthma and other inflammatory respiratory or skin disorders. In addition, some halophilic archaea are known to produce metabolites that may be useful for the treatment of cancer (Kowalewicz‐Kulbat et al. 2023).

The in‐depth characterisation of the isolated halophilic archaea will show which strain(s) will be most interesting for immune‐modulatory purposes and as sources for important metabolites with anti‐bacterial or anti‐cancer properties. It will also be interesting to compare the microbial profile of the air to those of the rocks in the same sampling site.

The limitations of the study include the fact that only cultivable micro‐organisms were analysed here, which does not reflect, but most likely underestimates the true diversity of the microbial communities in these sites. Comprehensive metagenomic studies would be required to describe the complete microbial diversity in the Bochnia Salt Mine, which was not the aim of this study. Furthermore, only micro‐organisms collected in the summer season were analysed here. We cannot exclude that the microbial communities may vary between seasons. Therefore, we plan to conduct a similar study on the microbial communities present during the winter season in the Bochnia Salt Mine. Finally, this study only refers to the Bochnia Salt Mine. It may be interesting to compare the microbial communities from the Bochnia Salt Mine to those from other salt mines used for halotherapy or salt mines not used for halotherapy.

## Author Contributions

Conceptualisation: Aleksandra Puławska, Magdalena Kowalewicz‐Kulbat, Camille Locht, Maciej Manecki. Funding acquisition: Aleksandra Puławska, Maciej Manecki, Luciana Albuquerque, Camille Locht, Magdalena Kowalewicz‐Kulbat. Performed the experiments: Aleksandra Puławska, Magdalena Kowalewicz‐Kulbat, Jolanta Kalinowska, Michalina Rachubik, Dominika Drzewiecka, Luciana Albuquerque. Analysed the data: Aleksandra Puławska, Magdalena Kowalewicz‐Kulbat, Jolanta Kalinowska, Luciana Albuquerque, Conceiçao Egas, Maciej Manecki, Camille Locht. Project administration: Aleksandra Puławska. Visualisation: Aleksandra Puławska, Krzysztof Krawczyk. Contributed reagents/materials: Aleksandra Puławska, Magdalena Kowalewicz‐Kulbat. Writing – original draft preparation, Camille Locht, Magdalena Kowalewicz‐Kulbat. Writing – review and editing, Aleksandra Puławska, Magdalena Kowalewicz‐Kulbat, Camille Locht. All authors read and approved the manuscript.

## Conflicts of Interest

The authors declare no conflicts of interest.

## Supporting information


**Data S1.** Supporting Information.

## Data Availability

The data that support the findings of this study are available on request from the corresponding author. The data are not publicly available due to privacy or ethical restrictions.
