# Supplementary material for: Halophilic and Non‐Halophilic Microbial Communities in Relation to Physico‐Chemical Characteristics of Salt Mine Air
Source: Environ Microbiol Rep. 2025 Apr 30;17(3):e70095. doi: 10.1111/1758-2229.70095 (PMC12042213; doi:10.1111/1758-2229.70095)
Supplement: Supplementary file 1 — Data S1. Supporting Information. [file EMI4-17-e70095-s001.docx]

**SUPPLEMENTARY MATERIAL**

Total suspended particulate matter (TSP) was collected on quartz filters (Whatman QMA, ϕ25 mm) using battery-powered personal air samplers (Gilian pumps, Sensidyne, USA). The filtered air volume was approximately 5 m³. Dreschler samplers were used for wet sampling to determine ion chemical composition in a liquid aerosol. Two scrubbers filled with 50 ml of ultrapure water were connected to the Gilian air pump, and 120 L of air was pumped through the water. One of the two obtained solutions at each sampling site was acidified using 0.5 mL ultrapure nitric acid (for cation analysis). Air temperature and relative humidity (RH) were determined using an Assmann psychrometer at each sampling point.

To study the relative proportions of halophilic organisms compared to other microorganisms in the four sampling sites, 100 L of air/sample site was aspirated using the impaction method with the MAS-100 Eco® air sampler (Merck) directly onto plates containing Tryptic Soy Agar (TSA) culture medium (BioMaxima, Lublin, Poland) (for non-halophilic microorganisms) or *Halobacterium* medium (HBM) containing 5 g/L yeast extract, 5 g/L casamino acids, 1 g/L Na-glutamate, 2 g/L KCl, 3 g/L Na_3_-citrate, 20 g/L MgSO_4_·7 H_2_O, 36 mg/L FeCl_2_·4 H_2_O, 360 ng/L MnCl_2_·4 H_2_O, 20 g agar and 15%, 20% or 25% NaCl (for the halophilic microorganisms). Each sampling was performed in triplicate. The TSA agar plates were then incubated for 10 days at 21°C, 28°C or 37°C, while the HBM agar plates were incubated for up to 3 months also at 21°C, 28°C or 37°C to determine the colony-forming units (CFU)/m^3^

**SUPPLEMENTARY TABLES**

Table S1. The concentration of ions in liquid aerosol and total suspended particulates (TSP) in the air of Bochnia Salt Mine at the indicated sampling sites.

|  | **BL-1** | **BL-2** | **BL-3** | **BL-4** |
| --- | --- | --- | --- | --- |
|  | Mean ± SD [µg/m^3^] | | | |
| Na^+^ | 214.3 ± 18 | 379.9 ±51 | 511.5 ± 57 | 1,196.9 ± 141 |
| Mg^2+^ | 7.9 ± 9 | 21.2 ± 4 | 38.9 ± 12 | 54.6 ± 7 |
| K^+^ | 23.1 ± 36 | 16.5 ± 4 | 26.1 ± 8 | 54.6 ± 17 |
| Ca^2+^ | 47.0 ± 64 | 64.1 ± 11 | 83.5 ± 21 | 218.2 ± 25 |
| F^-^ | 49.3 ± 44 | 83.3 ± 10 | 44.7 ± 11 | 73.7 ± 7 |
| Cl^-^ | 452.1 ± 27 | 576.3 ± 74 | 701.8 ± 92 | 1,468.7 ± 67 |
| PO_4_^3-^ | 8.9 ± 19 | n.d. | 14.4 ± 28 | n.d. |
| SO_4_^2-^ | 144.4 ± 16 | 131.0 ± 8 | 91.8 ± 18 | 327.3 ± 55 |
| Al^3+^ | 7.1 ± 5 | 26.4 ± 13 | 36.2 ± 45 | 5.1 ± 1 |
| Fe^3+^ | 51.8 ± 15 | 44.7 ± 24 | 42.7 ± 23 | 59.1 ± 15 |
| Mn^2+^ | n.d. | 3.3 ± 5 | 3.1 ± 4 | 1.7 ± 2 |
| Cr^3+^ | 2.0 ± 3 | 0.1 ± 0.3 | 2.5 ± 4 | 1.1 ± 1 |
| Cu^2+^ | 0.7 ± 1 | 1.1 ± 2 | 1.6 ± 2 | 1.2 ± 2 |
| Zn^2+^ | 7.2 ± 9 | 4.3 ± 3 | 10.0 ± 6 | 4.0 ± 4 |
| TSP | 23.4 ± 10 | 16.5 ± 5 | 222.5 ± 22 | 21.6 ± 6 |

n.d.- values below limit of detection

Table S2. Concentration of carbonaceous fraction, water-soluble fraction, and microelements associated with TSP in the air of the Bochnia Salt Mine at indicated sampling sites.

|  | **BL-1** | **BL-2** | **BL-3** | **BL-4** |  | **BL-1** | **BL-2** | **BL-3** | **BL-4** |
| --- | --- | --- | --- | --- | --- | --- | --- | --- | --- |
|  | Carbonaceous and water-soluble fraction [µg/m^3^] | | | |  | Microelements  [ng/m^3^] | | | |
| **OC** | 4.15 | 1.92 | 19.72 | 4.53 | **Al** | 304 | 744 | 1804 | 695 |
| **EC** | 0.41 | 0.18 | 0.91 | 0.17 | **Fe** | 374 | 864 | 3,204 | 4,831 |
| **TC** | 4.56 | 2.10 | 20.63 | 4.71 | **Mn** | 16 | 26 | 82 | 213 |
| **Na^+^** | n.d. | 0.08 | 5.52 | 0.14 | **Ti** | 239 | 309 | 377 | 273 |
| **NH_4_^+^** | 0.43 | 0.45 | n.d. | 0.30 | **As** | n.d. | n.d. | n.d. | n.d. |
| **Mg^2+^** | n.d. | n.d. | n.d. | n.d. | **Ba** | 13 | 18 | 45 | 20 |
| **K^+^** | n.d. | n.d. | 0.93 | n.d. | **Cr** | n.d. | n.d. | n.d. | n.d. |
| **Ca^2+^** | 0.14 | 0.14 | 4.14 | 0.70 | **Cu** | 15 | 19 | 43 | 30 |
| **F^-^** | n.d. | n.d. | 0.21 | n.d. | **Mo** | n.d. | n.d. | n.d. | n.d. |
| **Cl^-^** | 0.02 | 0.71 | 13.33 | 0.67 | **Ni** | 13 | n.d. | n.d. | n.d. |
| **NO_2_-** | n.d. | n.d. | n.d. | n.d. | **Pb** | 2 | n.d. | n.d. | n.d. |
| **Br^-^** | n.d. | n.d. | 0.07 | n.d. | **Sr** | 7 | 7 | 35 | 11 |
| **NO_3_-** | 0.78 | 0.11 | 0.09 | 0.01 | **Zn** | 63 | 85 | 130 | 73 |
| **PO_4_^3-^** | 0.07 | 0.03 | n.d. | 0.04 |  |  |  |  |  |
| **SO_4_^2-^** | 1.44 | 1.59 | 7.14 | 2.53 |  |  |  |  |  |

n.d.- values below limit of detection; OC, organic carbon; EC, elemental carbon; TC, total carbon.
